# Limitless capacity: a dynamic object-oriented approach to short-term memory

**DOI:** 10.3389/fpsyg.2015.00293

**Published:** 2015-03-23

**Authors:** Bill Macken, John Taylor, Dylan Jones

**Affiliations:** School of Psychology, Cardiff UniversityCardiff, UK

**Keywords:** short-term memory, limited capacity, perceptual-motor processing, perceptual organization, language and memory

## Abstract

The notion of capacity-limited processing systems is a core element of cognitive accounts of limited and variable performance, enshrined within the short-term memory construct. We begin with a detailed critical analysis of the conceptual bases of this view and argue that there are fundamental problems – ones that go to the heart of cognitivism more generally – that render it untenable. In place of limited capacity systems, we propose a framework for explaining performance that focuses on the dynamic interplay of three aspects of any given setting: the particular task that must be accomplished, the nature and form of the material upon which the task must be performed, and the repertoire of skills and perceptual-motor functions possessed by the participant. We provide empirical examples of the applications of this framework in areas of performance typically accounted for by reference to capacity-limited short-term memory processes.

It is paradigmatic in cognitive psychology to attribute performance limitations, in the final instance, to the limited capacity of the processing systems that underpin that performance. This is particularly the case in short-term memory, a 60-years old paradigm in which the profound difference between these two concepts – contextually limited performance and structurally limited processing capacity – is not often confronted or even acknowledged. Indeed, the idea of a limited-capacity, short-term memory system appears as an integral part in explanations of aspects of cognition as broad and diverse as the development and evolution of language (e.g., Baddeley et al., 1998; Wray, 2000), individual differences in intelligence (Hornung et al., 2011), distraction from task-irrelevant material (e.g., Lavie, 2005), mental arithmetic (e.g., Lee and Kang, 2002) and logical reasoning (e.g., Gilhooly, 2004). So embedded is this explanatory device that limited performance and limited capacity present themselves as inseparable, even identical, postulates. Here, we aim to scrutinize the conceptual and empirical underpinnings of these postulates, and we end by rejecting both of them as either determinable or veridical aspects of human functioning.

We do this from the perspective of what has become known as ‘embodied’ or ‘grounded’ cognition, which is to say that our explanatory concepts stem from a focus on the corporeal organism interacting adaptively with its environment. The components of that interaction are the sensory-motor processes of the organism and the way in which they enable it to gather information about and interact dynamically with its ecology. There are a vast range of projects and approaches that fall under the general term ‘embodiment’ (for broad overviews, see e.g., Glenberg, 1997; Wilson, 2002; Barsalou, 2008; Lakoff, 2012), but here, the relevance stems from our attempt to provide an alternative account for phenomena that classical cognitive science has sought to explain in terms of processes (e.g., encoding, storage, decay, interference) operating on ‘central’ representations whose essential form transcends the perceptual processes whereby they may be transduced and the motor processes whereby they may be converted into actions. We begin to pose our alternative, embodied approach by considering the basis of the foundational ideas of capacity limitation in cognitive science and set it within a broader, general critique of cognition.

## Capacity Limitation and the Genesis of Cognitive Psychology

When the crisis of Behaviorism occurred in the psychology of the 1950s, key advances in a range of domains provided a context for new thinking about human behavior. In particular, in a decade that was to shape human endeavor in many ways, a range of ideas which could be readily applied to an understanding of human performance were those about information processing and the programmable digital computer. There are two interconnected aspects of these ideas that provide the foundational basis for cognitive psychology: first, quanta of information can be posited, and, second, there are limits to the number of quanta that can be processed at any given moment (e.g., Shannon and Weaver, 1949; Turing, 1950). From this perspective, the limited performance of a device derives ultimately from the limited capacity for information transmission of its basic processing systems.

If the basis of intelligent behavior is the manipulation and transformation of such quanta of information (e.g., Newell, 1990), then it is necessary to establish what that quantum is in a given setting. Cognitive psychology construes capacity limitation in a variety of ways. For some, it is construed as structurally limited ‘slots’ for the representation of information (e.g., Luck and Vogel, 1997). For others, it manifests as finite processing resources that must be allocated over units of information (e.g., Cowan, 1995; Bays and Husain, 2008), or as temporal constraints on the maintenance of certain types of information (e.g., Baddeley et al., 1975; Barrouillet et al., 2011). Others model capacity limitation in terms of interactions between representations of different elements of information leading to interference or displacement (Lewandowsky and Oberauer, 2009). Others incorporate more than one such conception. Our critique here applies to all of these approaches as they all, in positing capacity-limited processing systems, share a fundamental underlying assumption; the very idea of capacity limitation necessarily connotes some primordial unit to which that capacity relates and by which it can be determined. Most commonly, in relation to capacity, these units are thought of as *items* or *chunks* (e.g., Miller, 1956; Newell and Simon, 1976; Cowan, 2000). However, while the *item* or *chunk* is conceived of as the basic unit to which processing is addressed, its dimensions or content are not necessarily the same in all settings; for a given process, a newly-learned multisyllabic word may be seen as an item formed of pre-existing syllables, but at the same time, those syllables may be deconstructed into smaller elements, such as phonemes.

So, even though they form a basic *sine qua non* for the idea that intelligent behavior may be thought of as the manipulation of units of information, what those elements might actually be – how they are to be quantized and quantified – is already far from clear. Cognition sidesteps this problem in operational terms by asserting what the experimenter or modeler judges to be the basic unit of any given task setting. Modeling attempts, while often explicitly remaining agnostic as to what the actual unit is, nonetheless proceed by asserting what it should be. So, within theories of short-term memory (e.g., Page and Norris, 1998; Burgess and Hitch, 1999; Henson, 1999), it may be a syllable or word, defined by a set of abstract features (vector values which themselves might be conceived of as primordial, or perhaps their constituent dimensions should be, and so on). The issue is exemplified in generative linguistics. Here, a formal and explicit attempt is made to define the primordial elements of a speech act in terms of phonological features that are central, in that they are meant to map to perception and execution while transcending both. So, for example, Chomsky and Halle (1968) posit a finite (provisional) set of phonological features from which all utterances may be assembled. This is just one example of the attempt to determine the basic units to which processing is addressed; in general terms, however, the endeavor pervades cognitive psychology, most assiduously perhaps in those attempts at quantifying capacity, where consideration may be given to whether, for example, a red circle should be deemed an item, or whether the critical quantities relate separately to its shape, its color, its location, and so on (e.g., Hardman and Cowan, 2014).

The problem here is knowing where to stop in the super- or sub-ordinate direction on any other than arbitrary (or arbitrarily expedient) grounds. More critically, by whatever means the determination of the units is achieved, a fundamental issue remains, which is that the problem that the cognitive system is meant to solve in order to support coherent behavior must disappear at some level of granularity, and if the problem can disappear at that level of granularity, why not at any other? Take, for example, the classic ‘problem of serial order’ (Lashley, 1951), a problem to which much formal theorizing about limited capacity short-term memory addresses itself. The problem of serial order is that since an organism’s behavior is temporally extended, the organism must implement some process whereby appropriate elements of that behavior are executed in the appropriate order. However, if it is indeed the case that a fundamental problem of behavior is that of the serial order of units of behavior, then there must necessarily be a point of granularity in behavior at which it is no longer a problem. This is so, simply because to posit the problem in this way means that there must be some primordial, indissoluble unit to which the ordering process is applied, otherwise one finds oneself, *à la* Zeno, in a regression to infinitesimally smaller levels of granularity. Thus, this conceptual approach to accounting for temporally extended behavior has to posit a realm where time does not exist (see Port and Leary, 2005). As with serial order, so with any other cognitive process: if there are *a priori* units which are the objects of constrained information processing, then those units must exist outside those constraints and must themselves already be formed, fully and indissolubly.

## The Nature of Symbolic Technologies and the Nature of Human Thought

The project of limited-capacity short-term memory, therefore, rests logically and methodologically on the possibility of positing *a priori* quanta that are essentially discrete and static: if they are not discrete, then this implicates basic quanta at either sub- or superordinate levels (which is to say that the *a priori* quanta for assessing capacity have been inappropriately defined), while if they are not static, then they are not amenable to the quantification on which the idea of capacity limitation necessarily rests. Before proposing an alternative to this general approach, we first consider why it is that human thought has come to be conceptualized in these cognitivist terms, and why an alternative conceptualization is necessary.

We argue here (see also Port, 2007; Wray, 2014) that the communicative technologies (of which writing is a prime example) developed by humans exert a profound influence on our intuitions, even as scientists, about the content and constituents of our behavior. The accomplishments of *Homo Sapiens,* and those that distinguish us from other animals, are less to do with the peculiarity or complexity of our behavior and our mastery over our environment than with the fact that we have developed symbolic technologies that serve to represent and organize that behavior. The products of the medieval cathedral builders are not so much distinguished from those of desert termites with respect to their architectural virtuosity, but in the fact that those cathedrals were first represented conceptually in a symbolic form – a plan – that could serve to coordinate the various inputs to those accomplishments (e.g., Marx, 1844/2007). Critical for current concerns, for about 6000 years human societies have been elaborating technologies for representing the meaningful contents of acts of speaking. The key feature of these technologies for our argument here, and that underlying their efficiency and survival, is that they comprise a finite set of discrete, static, *a priori* elements – symbols that may be assigned to the putative constituent sounds of speech – that can be lawfully combined in different ways to represent the utterances produced within a language. From early Phoenician attempts, through to the International Phonetic Alphabet we witness symbolic technologies that represent speech as an assembly of serially ordered segments that are discrete and static. As with written language, so too the technologies of logic and mathematics: they have been elaborated and refined to provide ever more powerful and abstract means of thinking about nature in terms of the lawful combination and transformation of symbols.

However, *prime facie* motivation for favoring an embodied over a traditional cognitivist approach to human thought arises because, unlike the static and discrete nature of symbolic technologies, biological systems and processes are inherently variable, graded and continuous in time and space, and so translating the behavior of those systems into descriptions based on the manipulation of discrete, static symbols necessarily loses something of the essence of those systems. In itself this is not a problem and may even be a necessity; analytic methodologies of whatever stripe have progressed by developing technologies for ‘freezing’ this infinite gradation and variability, be it via the mathematics of calculus or the phonetic transcription of an utterance. Furthermore, if the gradation and variability that characterizes the observable behavior of biological systems were merely a surface aspect either of our measurement processes or of the noisy contingencies of any particular instance of behavior (just such an assumption lies at the heart of the generative approach to language), then again, the translation from graded and variable to discrete and static would pose no particular underlying issue. However, the development of dynamic systems theory, and a body of evidence that would not have been obtainable without the developments in computing power in recent decades, have revealed that, far from being contingent or epiphenomenal, this graded, variable nature of biological systems is precisely the essential characteristic that enables them to give rise to new forms through phylogenetic and ontogenetic development (e.g., Thelen and Smith, 1994).

As with biological systems generally, so too with language, where the massive variability in actually perceived and produced language – variability arising from contextual factors ranging from the level of the utterance, to that of the individual physical and environmental heritage, to that of the history of a particular linguistic community – serves (while posing a challenge for the language learner) as the engine for development, both in the particular phonological form of a given language and in the acquisition of that language by any individual learner (e.g., Pierrehumbert, 2003; Port, 2010). It is unsurprising, from this perspective, that the technologies designed to capture spoken language – based on the lawful combination of a finite set of static segments – obscure many of its inherent characteristics and functions. In this respect, the disjunctions between linguistic technologies and speech are manifold and well-known, from the famous elusiveness of invariant phonemes in actual speech, to the deliquescence under critical scrutiny of things whose existence seems as self-evident as ‘words’ (Wray, 2014), and the remarkably formulaic behavior of utterances that, to the literate, appear readily analyzable and manipulable (Goldberg, 2003; See Beckner et al., 2009 for an overview).

So, while our symbolic technologies for studying nature are, by design, static and discrete, that which we use them to understand is variable and graded. There should be nothing controversial in the observation that these symbolic technologies are not the same as the things to which they apply. We maintain, however, that so immersed are we, not only in the use of speech and in its formal segmental representation in writing, but also in the process of translating from one to the other and back again, that the nature of the technology for representing the thing comes to appear to us as constituting the essential nature of the thing itself. Indeed, many of our intuitions about the nature of speech turn out to derive from conventions of literacy training rather than inherent essential properties of speech sounds or acts (Port, 2007; Wray, 2014). As such, we argue, the cognitivist conception of human thought constitutes a reification – one which an embodied approach has the potential to overcome – whereby those symbolic technologies that are used to represent our thought processes are taken to embody the essential nature of those processes.

A host of conceptual problems can be traced to this reification, not least the essential idea in cognition that any act is the end result of a mental plan that is both precursor to and prefigurative of that act. While some older critiques of such ideas may not receive much current consideration (e.g., Ryle, 1949), more contemporary ones continue to gain purchase (e.g., Thelen and Smith, 1994). The outcome on which we are focused here, however, is that the conflation of the technology with the process of interest – exemplified here by language – leads to the sense that the process itself involves the lawful assembly and manipulation of discrete, static *a priori* units, rather than being inherently graded, variable and dynamic. However, a particular characteristic of the typical conduct of cognitive science serves to circumvent, or at least obscure, such problems; the material input and output of the process under investigation is *already* operationally quantized and ordered, and so the relationship between those inputs and outputs which forms the basis of inference about the processes whereby the inputs are transformed to outputs will necessarily afford description in quantized and ordered terms.

A clear illustration of the distinction between approaching a process from a traditional cognitivist versus an embodied approach can be seen in relation to the study of speech errors that plays a key role in theories of speech production generally. Importantly, the patterns of such errors is typically taken to provide strong support for the idea that speaking involves the lawful assembly of segments into extended utterances according to syntactic constraints across lexical, sub-lexical, and supra-lexical levels. (see e.g., Meyer, 1992; Dell et al., 1993; Levelt, 1993). However, as has been noted elsewhere (Port, 2010) the transcription of utterances into an ordered series of discrete tokens – transcription that provides the basis for the analysis of error patterns – means that the data, while it can reveal different kinds of constraints on segmental order errors, can *only* reveal segmental order errors, and therefore the possible accounts of those errors is already constrained to those based on the ordering of discrete segments, excluding variations of other kinds.

The problem with this becomes evident when speech errors are examined instead through real-time measurement of vocal tract movement during speaking (e.g., Goldstein et al., 2007). Such studies reveal that speech errors, like other types of errors in movement, reflect outcomes of simultaneous and graded execution of more than one gesture at any moment, rather than erroneous and exclusive assignment of segments to ordered frames. For example, when required to repeatedly utter the pair ‘cop–top,’ people occasionally make errors such as, for example saying ‘cop–cop.’ In a segmental transcription this error can only be one of two things – either the exclusive substitution of the /k/ for the /t/ at the onset of the second syllable, or the exclusive substitution of the word ‘cop’ for the word ‘top’; either way, the error conforms to a scheme whereby segments are assembled into ordered frames. However, the actual movement of the vocal tract reveals gradations in the errors, such that the dorsum of the tongue (the place of articulation for /k/) varies in height at the point in time when the speaker should be saying the onset of ‘top,’ involving the tip of the tongue. In most cases it remains low but sometimes it achieves a height beyond that which would be appropriate for execution of the target syllable ‘top.’ Importantly, not only do these errors vary continuously (i.e., the dorsum does not just bimodally occupy erroneous ‘up’ or correct ‘down’ positions), but the erroneous movement of the dorsum is sometimes executed simultaneously with the correct target movement of the tip of the tongue to execute the /t/. The variation is continuous, not discrete or categorical. Furthermore, when these movement errors are compared with those present in phonetic transcription of the utterances, many of them turn out not to be so recorded. Detailed real-time analysis, therefore, reveals a picture in which errors of speech involve the simultaneous, graded and continuous real-time execution of more than one articulatory gesture, some of which may be captured by the discrete, ordered phonetic transcription and some of which are not.

The key point here is that the methodology of converting behavior into a discrete, ordered code not only obscures the graded and continuous nature of behavior, but ensures that explanations of that behavior in terms of discrete ordered segments are the only ones that present themselves. Here the conflation of behavior with the technology for representing it not only engenders a particular way of construing the essence of that behavior, but also means that the evidence collected about that behavior comes in a form that necessarily accords with the particular paradigmatic assumptions – that is, that the behavior of interest can be understood in terms of the processing of discrete, static units – and so will never cast doubt, in itself, on the appropriateness of that very paradigm. This precise problem exists in relation to capacity: if we performatively determine what the primordial units of processing are, and we implement those units in the input and chronicle their appearance or otherwise in the output, not only may we never quite settle on a precise determination of what the capacity of the system is (and the literature has not, to date, settled on such), neither will we ever see evidence that we are fundamentally misconstruing the nature of the process we are trying to understand. So, the ongoing theoretical debate about the capacity of short-term memory focuses on questions about what the primordial unit is and how it is to be operationally observed (items, features, chunks, etc.), how many different types of unit there are (verbal, non-verbal, spatial, visual, etc.), and how many and what type of systems there are for processing each of them.

## Dissolving the Question of Capacity: Dynamic Objects Versus Static Items

**FIGURE 1:**
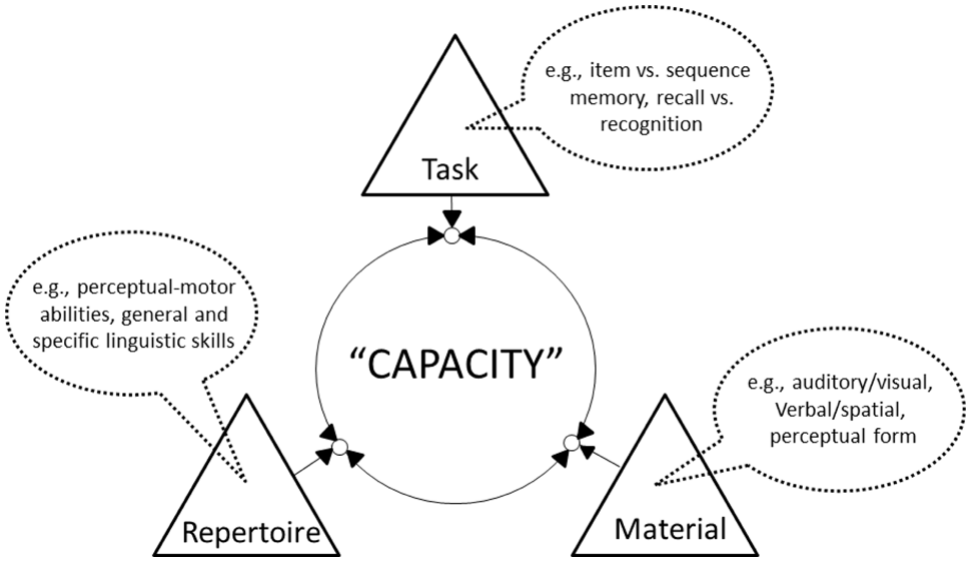
**Schematic illustration of the framework, within which performance is a dynamic outcome of three broad classes of factor, each of which may modify the influence of each of the others**.

Rather than seeking explanations of limited performance within the structural characteristics of purported systems and representations underpinning that performance, we instead advocate a framework that focuses on the dynamic interplay of three broad aspects of any given performance setting (see **Figure 1** for a schematic representation), none of which alone can be said to be determinate of performance. The first aspect of the setting is the particular *task* that must be accomplished. This involves not merely the attribution of a function such as ‘short-term memory’ to a particular task, but a specific consideration of the precise requirements of process. So, for example, serial *recall*, involving the reproduction of a sequence after a brief interval, is fundamentally different from serial *recognition* within which no such reproduction is required. A second aspect is the nature and form of the *material* upon which the particular task must be performed. Here too, consideration must go beyond mere questions of content – e.g., verbal versus visuospatial information – and must also address aspects of the formal organization of that particular content as it may impact on its perception. The third aspect is the available *repertoire* of the performer such as it may be deployed given the two preceding aspects. This repertoire is construed as including general and specific skills (e.g., the general ability for fluent speech production and the specific familiarity with a particular set of verbal material) as well as the performer’s perceptual and motor functions (for similar perspectives, see e.g., Craik and Lockhart, 1972; Ericsson and Kintsch, 1995).

The limit to performance – ‘capacity’ – within this framework is a direct consequence of the confluence of these three broad aspects, with greater congruence amongst them leading to better performance. In this way, our account draws on and elaborates upon the notion of affordance (e.g., Gibson, 1977; Norman, 1988). It shifts the explanatory role of perceptual-motor processing into the foreground, making it a central concern in explanations of short-term memory performance, rather than, as is more conventionally the case, assigning it to the realm of peripheral input/output processing.

None of these aspects of the setting is regarded here as determined, or indeed determinate, in an *a priori* way. Therefore, the explanatory framework we propose is immune from the type of problems discussed above with *a priori* specification of the nature of the items to be processed and the structural properties of the systems that do that processing that inhere in the traditional view of capacity-limited short-term memory. Our objective here, then, is not to resolve the question of capacity, but to supplant it. The key conceptual component of this is to replace the notion of an item as the explanatory unit, with the notion of an object. This is not an unproblematic notion, although the difficulties are fundamentally different to those that accompany the notion of an item. The problematic character of the object, as we use it here, stems from its inherently dynamic nature – it resides neither in the environment, nor in the eye of the beholder, nor in the particular task goals on a given occasion, but rather in the dynamic interplay amongst all three aspects. Undoubtedly, properties of perceptual systems – a function of both ontogenetic and phylogenetic adaptations – and the environment the organism encounters play a key role in the formation of objects, but as a functional unit of performance, the object is also constrained and modified by the particular task at hand. So, an object is brought into being momentarily by the characteristics of these three broad aspects and dissolves or mutates with changes in those aspects. In one setting, the object of performance may be a holistic, temporally extended sequence of sounds, while with a change in, for example, the acoustic properties of that sound, or in the task that has to be performed, the object is transformed into larger or smaller objects. We provide detailed instantiations of these ideas in following sections.

Objects are instantiated in both perceptual and motor forms, with the impact of obligatory and deliberate processes manifested differently in the two domains. Perceptual systems serve to organize information from the environment into objects in an obligatory way, although some environmental input – exemplified in vision by the Necker cube – is also amenable to deliberate reorganization (see e.g., Macken et al., 2003). Movement is also object-oriented in this sense, but here, deliberate, goal-directed action is implicated more typically, for example, in the selection of a particular manual grip configuration to use a tool for cutting as opposed to stabbing. This mapping of perceptual-obligatory and motor-deliberate is not exclusive. So, the obligatory processes of perceptual object formation may establish in a given environment (including both the material and the task) a set of affordances that may be obligatorily mapped onto systems for motor control. Thus, motor control systems are under a degree of obligatory access from perceptual systems (e.g., Rizzolatti and Luppino, 2001; Hickok and Poeppel, 2004). At the same time, the particular activity of motor control systems may influence the precise way in which the perceptual system organizes information in the environment (e.g., Ganel and Goodale, 2003). Thus, objects are formed dynamically out of the combined influence of the three broad aspects of the setting described above. Changes in any aspect may lead to changes in the form of these functional units. The level of performance achieved in any given setting is then a function of the readiness with which appropriate objects to accomplish the task may be formed, and obstacles to task performance are due either to impediments to the ready formation of appropriate objects or to the ready formation of objects that are inappropriate to the particular task requirements.

Below, we demonstrate the usefulness of this approach for understanding short-term memory performance by providing a detailed account of empirical findings to explicate how these concepts play out, and how they provide a coherent account of performance in short-term memory. In replacing the item with the object, our approach overcomes the problem of what the *a priori* units of processing are by not seeking to posit such units in the first place. Rather, we begin analytically by doing away with the assumption that there is any primordial unit, the manipulation of which underpins performance. Necessarily this requires methodological bootstrapping whereby we quantify performance under various conditions, but without actually insisting that the process of quantification addresses the essential units of that performance. The success or otherwise of this approach may be judged in the detailed account of empirical phenomena discussed below. Beyond this, in conceiving of the functional units of performance in momentary, dynamic terms, the question of deriving an underlying capacity limitation is obviated, since a change in any of the inputs to the dynamic system may fundamentally change the system’s performance in relation to the task requirements, and do so in an unlimited way. Throughout the following empirical examples, we illustrate these concepts in a concrete way, illustrating the superiority of such an account of performance that focuses on the specific nature of the task, the form and content of the material, and the repertoire of skills and perceptual-motor processes of the participant over one that seeks to invoke capacity-limited systems underpinning that performance.

## Language and Short-Term Memory: Repertoire, Material, and Task

We begin discussion of how these ideas play out empirically by considering the role of language in short-term memory performance, not only because it forms a key focus in theories of the structure and functions of putative short-term memory systems, but also because language presents itself as a cognitive system *par excellence*. Conventional approaches explain the role of linguistic familiarity in short-term memory (e.g., lexical frequency) through its effect in supporting stability and/or retrievability, via long-term representations, of the volatile short-term representations being processed within verbal short-term memory, thereby ameliorating some of the effects of limited capacity (see e.g., Hulme et al., 1991; Schweickert, 1993; Gathercole et al., 2001). Some deficits associated with this account are illustrated in a series of experiments that manipulates such familiarity with the verbal material (Woodward et al., 2008).

A typical experiment from that series involved short-term serial recall for sequences of non-words (i.e., ones which, at the outset, were novel to the participants). Twelve such non-words were divided into two sets (set A and set B, each of six non-words). The beginning of the procedure involved assessing participants’ skill (fluency) in saying aloud this verbal material. They were required to read from a computer screen, as quickly and accurately as possible, three types of presentation: single non-words, pairs of non-words and six-non-word sequences. For the pairs and sequences, the stimuli were either ‘pure,’ in that they were drawn solely from either the A or the B set, or ‘mixed,’ in that they were constructed from alternate non-words drawn from each set. While all participants bring their general fluent speaking ability to the setting, they were then familiarized with this material via 60 trials of serial recall for six-non-word sequences constructed of random orderings of A and B sets (30 trials for each set). Only ‘pure’ sequences were presented during this familiarization phase. Importantly, each of the 12 non-words was encountered equally often within this familiarization phase, and so all the individual items should become comparably familiar.

Following this phase, the measures of fluency described above were taken and compared with baseline skill measures. Familiarization with the material via the serial recall phase led to no reduction in spoken rate for either singles or pairs of non-words. However, the practice phase did enhance the fluency with which 6-non-word sequences were produced, indicated by a reduction in the time taken to speak aloud the sequences. Importantly, this enhancement was not merely due to practice with rehearsing and recalling sequences of this length, since the enhancement only occurred for ‘pure’ sequences with no change in the fluency with which ‘mixed’ sequences were produced as a result of the practice. This, then, indicates a very specific skill enhancement in this setting. What is more, when serial recall performance was subsequently tested, this specific skill enhancement led to superior recall performance for ‘pure’ versus ‘mixed’ lists, even though each type of list is constructed from items that have been encountered equally often during the familiarization phase.

Clearly, therefore, no explanation couched solely in terms of familiarity with supposed elements of the material (‘items’) or familiarity with the particular task (serial recall) can account for performance. It might be argued that the familiarization phase led to the establishment of representations of each item organized in associative networks defined by set, and that such associations provide support during recall which will be more evident for pure than mixed sequences (see e.g., Stuart and Hulme, 2000). However, in its focus on the maintenance and retrievability of volatile short-term representations, it is not clear that such a process could also account for the specific enhancement in fluency of speech output when the verbal material does not need to be recalled, but is merely read aloud from the screen. In other words, an account couched in traditional terms would need to suggest different mechanisms giving rise to the recall advantage and to the fluency advantage. On the other hand, an account which emphasizes the increased fluency in motor control associated with specific articulatory practice at assembling sequences of non-words from each of the two sets suffers no such lack of parsimony; the recall advantage and the fluency advantage are merely two consequences of the particular confluence of the task requirements, the nature of the material, and the specific skill within the participant’s repertoire.

Undoubtedly, while such evidence raises problems with the general conceptualisation of the role of language in short-term memory, in itself it does not necessarily undermine the notion of ‘central’ items and chunks which underpin theorizing about limited capacity systems. Indeed, such evidence of effects of experience on a range of skills (memory and fluency) fits readily with classical cognitive ideas about the formation of chunks from smaller entities as a function of practice (e.g., Newell and Simon, 1976). However, a number of other aspects of these effects seem to point more specifically to a motor – specifically co-articulatory – basis to the increased performance, rather than to the formation of larger chunks in central representations of the material. For example, the type of sequential practice described above with a set of non-words led to enhanced subsequent performance with a different set of non-words if that set contained the same articulatory offsets and onsets between non-words as the familiarized sets. However, no such transfer occurred for sets of non-words that shared the internal vowel segment with the practiced set (Woodward et al., 2008, Experiment 3). Similarly, if the familiarization task involved the practiced sequences being uttered in a paced fashion so as to prevent actual co-articulation of successive non-words, then the particular advantage for pure over mixed sequences did not emerge, even though each set of non-words had been encountered together in a practice sequence the same number of times (Woodward, unpublished).

Further non-trivial shortcomings in the classical cognitivist account of the role of language in short-term memory performance become evident when the detailed aspects of the task and the perceptual form of the material are examined. Key evidence for the classical, item-oriented, account of the influence of long-term linguistic knowledge on short-term memory relates to the different influence of such knowledge on serial *recall* and serial *recognition*; a robust influence of linguistic familiarity (specifically, lexicality) is found when performance is tested by serial recall, but that effect is significantly diminished or eliminated when performance involves serial recognition. The key functional distinction between these two tasks is that in recall, the requirement is to reproduce in some form the previously presented sequence, while in recognition, a sequence is presented, followed by a second sequence, identical to or slightly different from (e.g., two adjacent items may be transposed) the original, and the task requires a *same*/*different* judgment. The substantial superiority of recall for words over non-words, and the relative absence of this effect in recognition has been taken to point to a role for linguistic knowledge in supporting the short-term maintenance or retrieval of verbal items; by re-presenting the memory items in the test cue a role for processes involved in the short-term retention of information about those items is obviated. In this way, the interaction between lexicality (words/non-words) and task (serial recall/serial recognition) provides key evidence for specifically mnemonic process (retrieval, maintenance) operating on item-level representations in short-term memory (e.g., Baddeley, 2003; Jefferies et al., 2009).

However, since such accounts are typically focussed on processes operating on central representations (maintenance, decay, interference, retrieval, etc.), the question of modality in which the material is presented is one that, until recently, went overlooked; specifically, while serial recall tasks have commonly been presented in both auditory and visual forms, serial recognition has almost always been implemented auditorily. For this reason, theorizing about the role of linguistic familiarity in short-term memory represents a case study in the folly of a paradigm that that relegates perception and motor control to matters of mere input to and output from a central system, the operation of which gives rise to the essential aspects of performance. We argued above that a key aspect of the influence of familiarity with linguistic material resides in the readiness with which it can be assembled (e.g., via co-articulatory fluency) into an extended sequence of articulatory gestures of the type that may subsume the reproduction of a sequence required in serial recall tasks. Indeed, there is abundant evidence that the influence of familiarity with a set of verbal material is particularly evident in settings in which performance involves the production or reproduction of extended sequences, rather than individual words (e.g., Wright, 1979; Woodward et al., 2008; Bybee, 2010). As already noted, serial recognition does not involve the requirement for sequence reproduction.

Furthermore, and critically in this context, temporally extended auditory sequences may be processed as holistic auditory objects such that they afford matching with other auditory objects in a global fashion – that is, one that does not rely on identification and comparison of the ostensible constituent elements across the two sequences to be judged same or different. For example, classic studies on the perception of auditory sequences have shown that people are able to judge whether two rapid sequences of the same sounds (e.g., a tone, a buzz, a hiss and a click) are in the same or different order without being able to identify what the order of the sounds is, or indeed to identify, for different sequence pairs, which elements have been re-ordered. The ability to identify the constituent source of any differences emerges only when the sequences are presented at a sufficiently slow rate (around 4–5 items per second) as to allow for each successive sound to be verbally labeled (see Warren, 1999). As such, given certain acoustic properties (such as rate and spectral similarity), the sequence of sounds functions a single unit – an object – while changes in those acoustic properties enables its dissolution into smaller objects. As the compass of the object changes, the type of performance afforded varies, facilitating either global matching of sequences or identification of individual constituents of the sequence along with their order.

Given this, presenting a sequence at certain rates in auditory form affords sequence matching performance that is, strictly, only nominally based on sequential processing; rather, the task can be construed as one in which a single auditory object is compared with another. Serial recall, on the other hand, since it always requires at output the reproduction of a sequence, will be influenced by the readiness with which such a sequence may be assembled from the input material regardless of its presentation modality – readiness that is facilitated by linguistic familiarity (Wright, 1979; Woodward et al., 2008). From this perspective, the interaction between lexicality and task type emerges not, as the classical account has it, because of the different burden on item retention in recall versus recognition together with the increased robustness of such item retention for linguistically familiar material. Rather it is because auditory sequences may be processed as single objects, and so tasks, like serial recognition, that may be accomplished with such a perceptual form, based on global perceptual matching, will not exhibit effects due the readiness with which its constituents may be processed. We tested this idea via the simple extension of examining serial recognition and serial recall for both auditory and visual forms of presentation (Macken et al., 2014). Although presentation and retrieval conditions were held constant across modalities, sequential presentation of visual verbal materials in the same location does not afford the same object formation that auditory presentation does and so the burden on sequential processing of the constituents is increased in that setting relative to auditory presentation. We showed that while the effect of lexicality was present regardless of presentation modality when serial recall was required, in serial recognition it was also present with visually presented lists but absent for auditory lists. The classical account, in which the influence of linguistic familiarity operates via enhanced processing of item-level lexical or syllabic representations, cannot account for this interaction. According to that view the influence of linguistic familiarity is minimized in settings where the burden on item retention is minimized, that is in serial recognition where the items are re-presented and there is no requirement on the participant to reproduce the content of the sequence. This condition applies whether recognition is auditory or visual – both forms involve re-presentation of the original items; hence, the effect of lexicality of the material should be the same regardless of presentation modality. From the object-based perspective, the effect is absent in serial recognition only when the material is presented auditorily since such a form of presentation affords holistic object matching, thus minimizing the need for processes associated with the deliberate assembly of the material into a sequence. For visual presentation there is no such affordance; therefore, the items must be encoded individually and composed into a sub-vocal sequence, a process that is modulated by linguistic familiarity. That the effect does indeed reside within the speech motor system, rather than within enhanced processing of lexical items, *per se*, receives further support from the fact that the effect of lexicality in visual serial recognition is eliminated if the speech motor system is otherwise occupied during the task (by requiring the participant to repeatedly whisper the task-irrelevant sequence *1, 2, 3...* ; Macken et al., 2014, Experiment 2).

The influence of language on short-term memory is, therefore, a complex one. Certainly, it is one in which perceptual and motor processes play a critical role, but even that does not tell the complete story, since a detailed consideration of the precise task requirements is also necessary in order to fully delineate its influence. Language presents itself as a skill, as both a generic aspect of repertoire that enables the formation of speech motor sequences that allow for the reproduction of target sequences, as well as a more specific skill that relates to the enhanced readiness with which particular sets of verbal material may be reproduced. However, the role of motor skill may or may not be evident in performance; some task settings may be addressed on a purely perceptual basis, within which, if the perceptual repertoire of the participant affords it, effects of linguistic familiarity may be absent, while changes to the task or to the form of presentation of the material may bring them back into play. Critically, it is not clear how the traditional approach to capacity could encompass this complex picture. With auditory serial recognition, the functional unit of performance is the whole sequence, while with visual serial recognition, the character of the constituents, along with the sequence, determines performance. The items in one setting are not manifest in the other, but neither is their manifestation determined by the form of their presentation, since if the task requirements are changed, the lexical character of the material is manifest in performance regardless of modality. Discerning in this pattern the lineaments of a system whose inherent characteristic involves a limit to the number of items of a particular type that may be processed is, we argue, a theoretically futile project.

### Objects, Affordances, and Short-Term Memory

The way in which the objects rendered by the perceptual repertoire of the participant, and the extent to which those objects determine short-term memory performance, is also evident in another canonical aspect of short-term memory performance typically attributed to processes operating at the item level: the *talker variability effect* (e.g., Greene, 1991; Goldinger et al., 1991). Serial recall for random sequences of, for example, spoken digits presented in a single voice is superior to a sequence alternating between a male and female voice on successive digits. In terms of the capacity-limited processing of items, this is usually explained by reference to an additional burden placed on the encoding of those items (due to the need to represent the indexical information relating to voice in the limited capacity system or to the need to recode the variable input into a homogenous, canonical form), thereby taxing the limited capacity for storage and/or processing. It is possible, however, to reconstrue these findings within our embodied, object-oriented framework, without recourse either to the notion of item or the burden placed on the limited capacity storage system by its encoding.

This task requires serial reproduction (be it spoken, written, typed, etc.) of the presented sequence of digits. When presented in a single (say, female) voice, auditory perceptual processes obligatorily organize that input into a single relatively coherent sequential representation that matches the required output order of the material. In other words, the content and formal organization of the material affords its ready mapping onto a speech output (or rehearsal) sequence that accords with the requirements of the task. However, that formal organization is fundamentally changed when voices alternate, say between male and female, from one spoken digit to the next. Under these circumstances, perceptual organization partitions the input into two objects, one corresponding to each voice. A substantial body of work on auditory perception (e.g., Bregman, 1990; Warren, 1999) has shown that a key consequence of this process of object formation is that there is very little coherence across objects; for example, the ability to discern the relative ordering of elements belonging to different objects is very poor, compared to the ability to determine the ordering of elements within an object. Consequently, neither of the objects formed by the alternating voices maps readily onto the required output form, since they represent sequences of alternate digits in the input sequence. Thus, in alternating lists the degree to which the material affords ready mapping from the perceptual input to the motor output is reduced due to obligatory perceptual processes that organize the input into sequential representations based on acoustic similarity, in this case the pitch and spectral qualities of the voice (see Hughes et al., 2009). Performance is reduced, therefore, since the combination of the form of the material and the perceptual repertoire of the participant renders objects that are less appropriate to the task requirement than when that material is presented in an acoustically homogenous form.

This partitioning of serial alternating voices into two same-voice objects takes time to build up (see e.g., Bregman, 1990; Carlyon et al., 2001), a fact that can be exploited to show how object formation, not the capacity-limited processing of individual items, is responsible for the talker-variability effect. The key manipulation involves a lead-in – a sequence of to-be-ignored items (e.g., a countdown from 9 to 1) prior to, and at the same tempo as the memory list – in either single- or alternating-voice form (see Hughes et al., 2009). By allowing the build-up of object formation to take place prior to presentation of the to-be-remembered material, a lead-in of alternating voices should promote perceptual segregation by voice within the alternating to-be-remembered sequence, thereby reducing still further the affordance or mapping between input sequence and required output sequence. If item encoding were responsible, this manipulation would be expected, minimally, to have no effect, or to reduce the impact of voice alternation within the sequence by virtue of familiarization with the acoustic variability. However, the alternating voice lead-in causes further reduction in performance beyond that found with the basic alternating voice condition (Hughes et al., 2009).

This is not to say, however, that perceptual organization *alone* determines performance since if the task requirements are changed such that the same verbal material no longer needs to be retained in order, but only memory for the content is required, then the talker variability effect no longer emerges as it does when the requirement is for serial reproduction of the sequence (Hughes et al., 2011). This further illustrates that the specific interplay of the form and content of the material, the nature of the task that needs to be accomplished with that material, and the repertoire of the participant jointly determine performance in a way that cannot readily be explained by reference to structural properties of any of those individual aspects alone. Since all of these factors combine interdependently to determine the level of performance, again we see the fundamental problem with the project of limited capacity. We might frame the question rhetorically: what is the correct combination of task, material and participant repertoire to choose in order to accurately assay the capacity of the underlying system?

## Interference as Task-Irrelevant Affordance

These foregoing examples illustrate the application of our framework to settings in which only task-relevant material is presented to the participant. In our next example, we show how the same framework applies in settings in which task-relevant and task-irrelevant material is present. From the traditional, capacity-limited perspective, the presence of task-irrelevant material taxes the capacity-limited system via mechanisms such as depletion of resources or structural interference between representations of the relevant and irrelevant items, typically based on the similarity between the two (see e.g., Cowan, 1995; Kane and Engle, 2003; Lavie, 2005; Oberauer and Lewandowsky, 2008; Baddeley, 2012). Within cognitive science more broadly, the assumption that an observed reduction in performance in the presence of task-irrelevant material must be due to some process leading to the degradation of the item-level representations underpinning that performance is one that is as unscrutinised as the conflation of limited performance with limited capacity; rather, debate is focused on the precise mechanism – decay, interference, depleted processing resources, etc. – that leads to the degradation (e.g., Oberauer and Lewandowsky, 2008**).** Here we propose an alternative account of interference that does not rest on this assumption.

The shortcomings of the classical, item-focussed, cognitivist perspective become clear when considering the impact on performance of the presence of task-irrelevant sound during a short-term memory task. The presentation of such sound, which participants are instructed to ignore and on the contents of which they are never tested, during a serial recall task leads to substantial (typically on the order of 30–50%) disruption to serial recall performance (e.g., Colle and Welsh, 1976; Salamé and Baddeley, 1982; Ellermeier and Zimmer, 1997). The effect cannot be due to the depletion of limited processing resources; the size of the effect does not diminish with time, either over the duration of an experimental procedure or over days and weeks (Hellbrück et al., 1996; Jones et al., 1997b), nor is predictable sound less disruptive than unpredictable sound (Tremblay and Jones, 1998), nor is the degree of disruption related to any other measures of what are considered a participant’s ability to resist distraction (Beaman, 2004). Neither can the effect be attributed to structural interference between representations of irrelevant and relevant items; the sound need not be verbal, nor is its disruptive potency a function of its similarity to the task-relevant material (see Macken et al., 2009). There are nonetheless basic, acoustic characteristics that are necessary within the sound for the effect to emerge; the sound must be perceptually segmentable (e.g., due to the presence of silent intervals between successive sound tokens or the presence of rapid modulations in frequency and/or amplitude within continuous sound, such as are found in continuous speech), and each segmented entity must be different from the preceding one. Perceptually continuous sounds, or repetitions of an identical token do not cause disruption (Jones et al., 1993). In other words, the sound must constitute a sequence, of whatever content. However, again, this aspect does not, on its own, determine performance in the presence of such sound. If the task requirements are changed so that reproduction of the memory sequence is no longer required, while retaining the requirement to remember all the content, then performance is unaffected by the presence of task-irrelevant acoustic sequences. A task requiring reproduction of a sequence constituted of a random ordering of all but one of the digits 1 through 9 will be substantially disrupted by the presence of task-irrelevant auditory sequences. However, if precisely the same material is presented, in precisely the same way, but now the task requires identification of which of the digits was missing from that particular sequence, then no such disruption is evident (e.g., Jones and Macken, 1993; Beaman and Jones, 1998; Macken et al., 1999). This task still requires retention of the content of the sequence until the response is required, but the particular sequential order is no longer relevant (or useful) in accomplishing it. Clearly, then, the sound is not disrupting performance by somehow degrading the representations of the memory material, since it should otherwise affect any performance that depends on the retention of that material.

How does this pattern of disruption in the presence of task-irrelevant material fit within out framework? In our account of the talker variability effect, we argued that key details of that effect emerge from the way in which the acoustically variable form of the verbal material leads to obligatory perceptual organization that does not readily afford what the participant is required to do in the particular setting; it leads to the formation of two auditory objects, neither of which corresponds to the target sequence. The general point here is that, for task-relevant material, the greater the appropriateness of the momentary object, i.e., the affordance, or perceptual-motor congruence, between the input and output forms, the better performance will be. Precisely the same mechanisms, we argue, are at play when we examine the disruptive impact of task-irrelevant sound on the ability to reproduce task-relevant sequences. To the extent that such sound affords the same activity that is required of the task-relevant material – that is, sequential reproduction – then competing affordances are established within the setting that impact upon the ready accomplishment of the specific task goal. The impact does not operate by degrading the representation of the relevant material, but by providing an alternative object competing with the task-relevant sequence for control of the motor output process required to accomplish the task (analogous to effects of competing affordances on reaching and grasping in the visuo-motor domain, see e.g., Cisek, 2007). The serial recall task is one requiring the reproduction of a sequence, and the environment contains not only the task-relevant sequence, but other sequences, which via processes of obligatory perceptual organization render objects that represent potential, alternative candidates for control of the sequential motor system utilized to perform the focal task. Thus, when the requirement for sequential output, as embedded in serial recall, is removed from the task and all that is required is retention of the content, the presence in the setting of task-irrelevant sequential affordances is no longer of relevance for the accomplishment of that task and so performance proceeds unhindered. So, if the task does not require the construction of a sequential motor object in order to reproduce the sequence, but rather only requires retention of the content, there is no cost associated with the presence of task-irrelevant sequences.

**FIGURE 2:**
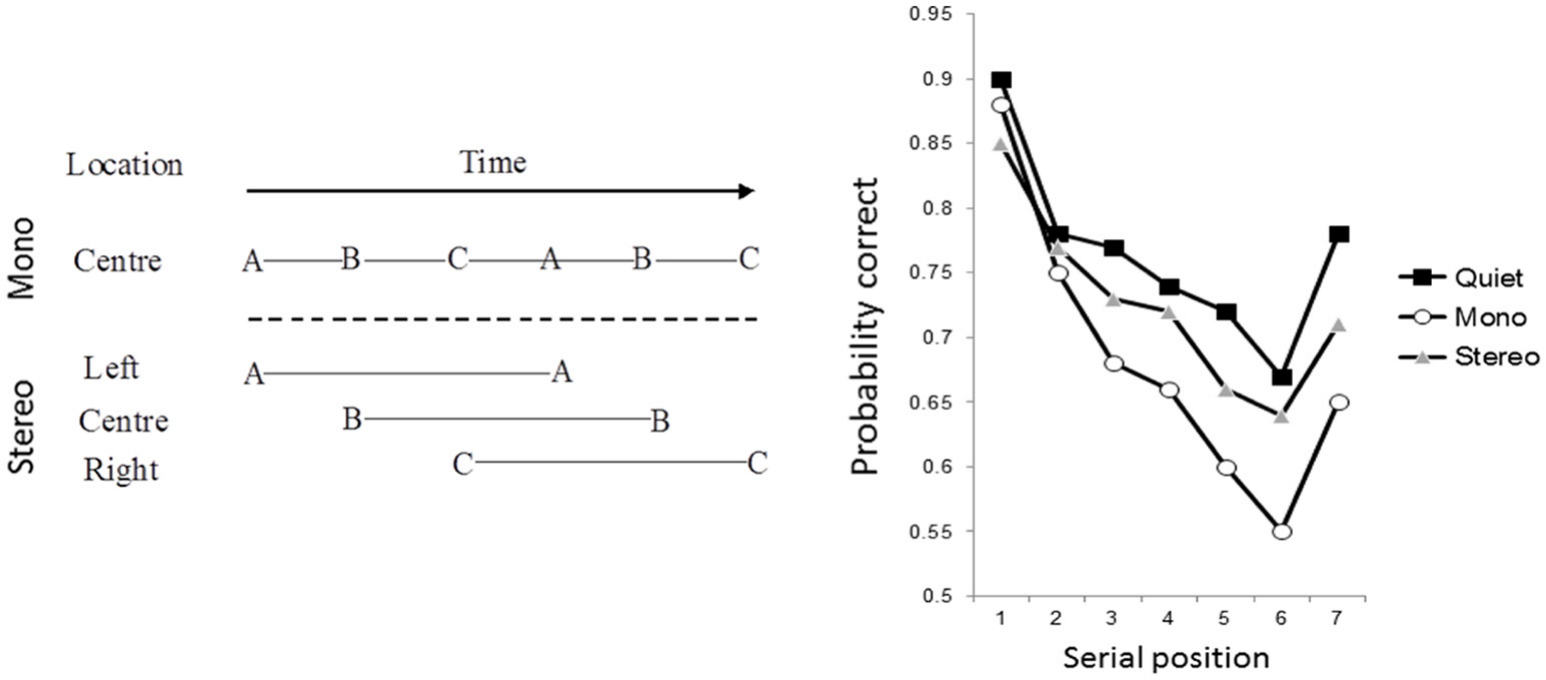
**The effect of the perceptual organization of task-irrelevant sound on its ability to disrupt serial recall**. The successive presentation of each of the three sounds (labeled *A, B,* and *C*) to the same location leads to the formation of a single, sequential object, competing with the target sequence. Assigning each successive sound to a different location leads to a form of organization in which the sound no longer contains sequential affordances.

Precisely the same de-potentiation of disruption from task-irrelevant material occurs if the sequential affordances are removed from that material. For example, Jones and Macken (1995) contrasted the disruptive effect of a repeated series of three sound tokens presented in a spatial configuration to give rise to perception of a single recurring sequence of three tokens (simultaneous stereophonic presentation via headphones) with that of the same three tokens, presented with the same recurring timing and order, but this time with each token presented from a separate location (left, center, and right channels of the headphones), thereby giving rise to the perception, not of a single sequence, but of three non-sequential streams of sound (see **Figure 2**). In the former case, the task-irrelevant material embodies sequential affordances, while in the latter, those affordances are stripped from the material by changing the form in which it is presented. This manipulation dramatically attenuates disruption, even though the content of the task-irrelevant material remains the same in both cases. So, here too, an account framed in terms of constituent items within task-irrelevant material competing with those within the task-relevant source for limited capacity storage or processing is untenable. The same content – the ostensible items – is present in both cases within the relevant and irrelevant material, but in one case the form of interaction leads to substantial impairment in performance, while in the other it does not.

## Interference as Object Formation

The power of this object-oriented approach in providing a better explanation of effects on performance classically attributed to interference becomes evident in two further, very different settings in which the ostensibly degrading effects of irrelevant on relevant items within capacity-limited systems have been charted. The first involves the effect of interpolated sound on the ability to make *same*/*different* judgments about the pitch of two successive tones, and the second involves the disruptive effect on serial recall of auditory sequences of a redundant auditory item occurring after sequence presentation.

**FIGURE 3:**
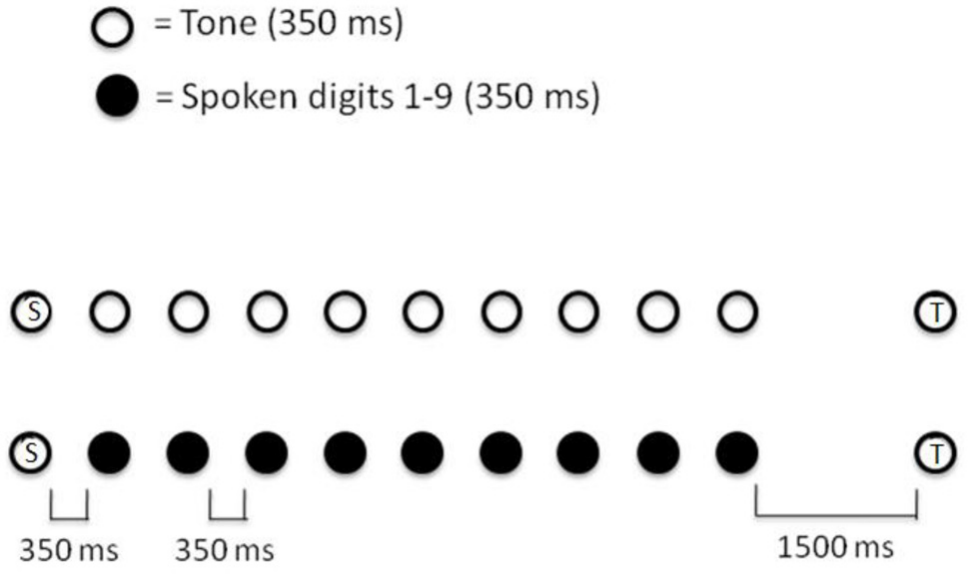
**Schematic depiction of the task from Deutsch (1970) and Jones et al. (1997a)**. The standard tone (S) is succeeded either by a series of other tones of varying frequency or by a random sequence of spoken digits, followed in turn by the test tone (T) either identical in pitch to the standard, or a semitone higher or lower.

A key focus for the debate about the source of limited capacity in short-term memory involves the ability to retain information about simple events in the presence of both variable time delays and the presence of different types of material interpolated between the initial presentation of that event and the requirement to make a judgment about it (e.g., Oberauer and Lewandowsky, 2008; Mercer and McKeown, 2010). **Figure 3** depicts such a setting in which it appears that performance is determined by interference amongst the representations of items within a limited capacity system. The setting, from Deutsch (1970) involves the presentation of a short tone – the standard – followed several seconds later by another tone – the test – either identical to or different from (plus/minus a semitone) the standard. Different types of to-be-ignored material may be interpolated between tones, and the task is to make a *same/different* judgment.

The basic finding is that performance on the pitch discrimination task is substantially poorer when the interpolated material comprises tones in a similar frequency range and with the same timbre as the standard and test tones than when that material is formed of spoken digits. On the face of it, this seems to provide clear evidence for similarity-based interference within capacity-limited storage; the representation of the standard tone is more subject to degradation by other, similar-sounding tones, than by the acoustically different digits. As such, by the time the test tone is presented, the information necessary to perform the task is still relatively intact in the latter compared to the former condition (e.g., Crowder, 1993). The capacity limits in the system underpinning the performance are therefore revealed by its inability to sustain a level of performance when it must represent other, similar, items to the target material compared to when no such irrelevant material is present, or when the irrelevant material is sufficiently different as to lead to no structural interference. However, a radically different account of this classic finding is provided by our object-oriented approach.

**FIGURE 4:**
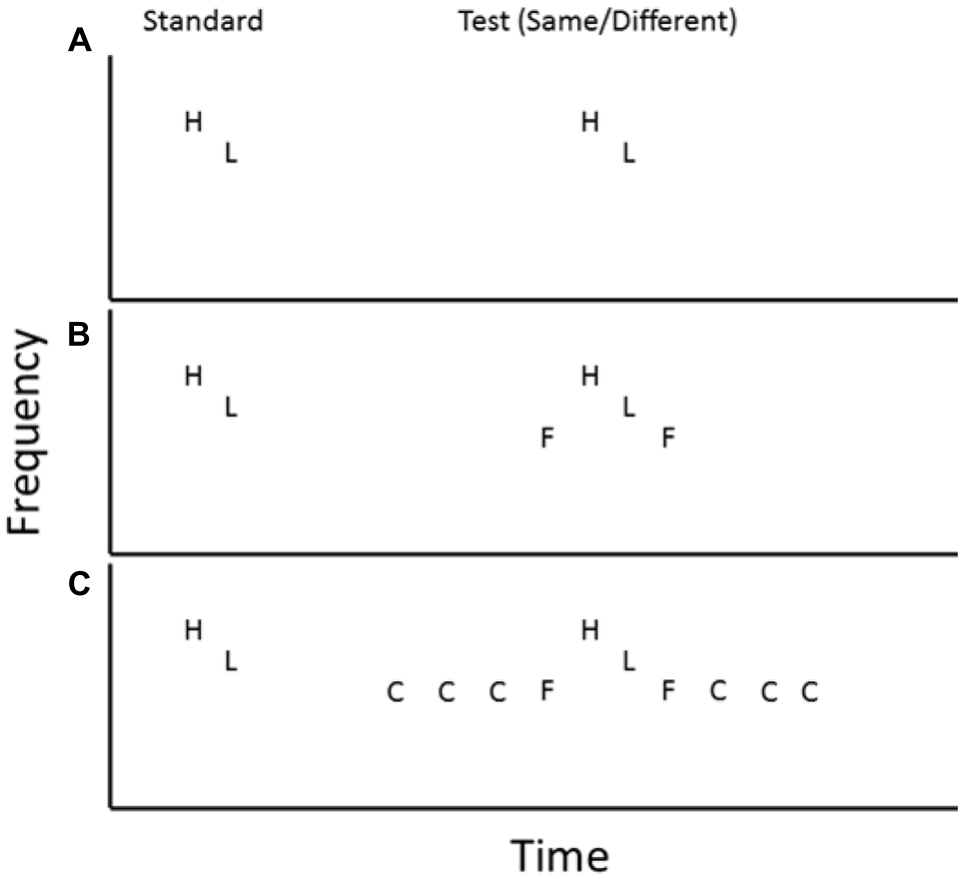
**Depiction of three critical conditions from Bregman and Rudnicky (1975)**. H, high tone, L, low tone, F, flanker tone, C, captor tone. Order of H and L tones may be either the same or different on both occasions. Panel **(A)** indicates condition where target test tones are presented on their own, panel **(B)** indicates the addition of flanker tones immediately before and after target test tones, and panel **(C)** indicates the further addition of captor tones preceding and following the flanker tones.

The starting point for this account is that the process of object-formation has implications for the addressability of the constituent features of that object. We touched on this in the discussion of the perceptual basis of auditory serial recognition, with respect to the findings that judgments about whether two auditory sequences are the same or different may be made even when access to the precise constituents that differ (or not) between the two sequences cannot be made (Warren, 1999). Thus, the formation of an auditory object from an extended sequence of sounds may impede the extent to which aspects of the segments of that object may be identified. A classic demonstration of this is depicted in **Figure 4**, in which the task is to judge whether the order of a pair of tones is the same or different on two instances (Bregman and Rudnicky, 1975). On the second instance, the target tones are either presented unaccompanied (**Figure 4A**) or in the presence of preceding and succeeding ‘flanker’ tones close in frequency to the targets (**Figure 4B**). Performance is markedly impaired under these conditions, but not because the flanker tones have somehow interfered with the representations of the target tones. That this cannot be the source of disruption is demonstrated in **Figure 4C** in which a series of ‘captor’ tones is presented before and after, and at the same frequency as, the flanker tones.

Under these circumstances, performance is restored to that observed when the targets are unaccompanied, although there is now even more potentially ‘interfering’ material in the acoustic setting. This pattern reveals the formation of different auditory objects across the three settings; in the first, the pairs of target tones in the standard and test stimuli form an initial and subsequent object that affords global matching in order to make the judgment. In **Figure 4B**, when the target tones are re-presented at test, they no longer form an object on their own, but rather are bound into a different object, along with the flanker tones, which does not afford ready matching with the standard stimulus, since the critical information needed to perform the task is now part of, and embedded within, the test object. The presentation of the captor tones serves to change the object formation again; in this case the flankers are bound into, on the basis of their identical frequency, an object with the captor tones. The identical acoustic composition of the captor and flanker tones forms a powerful perceptual cue to them forming a coherent object in its own right, leading to the perceptual segregation of the target material from the preceding and succeeding material. This isolation as an object once more permits ready comparison of standard and test stimuli.

**FIGURE 5:**
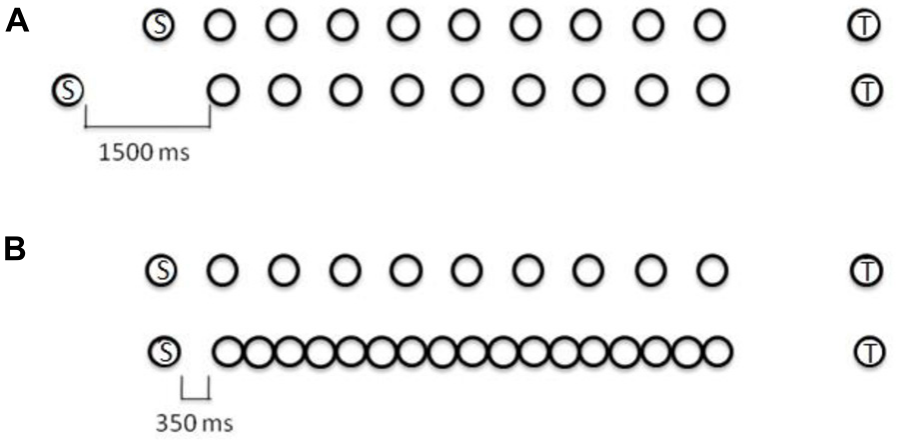
**Schematic depiction of two key manipulations from Jones et al. (1997a)**. Panel **(A)** illustrates the manipulation of increasing the interval between the standard and the first interpolated tone, while Panel **(B)** illustrates the manipulation of doubling the number and rate of interpolated tones. In both cases, performance is better in the conditions illustrated in lower stimulus type than the upper one.

It is not interference between item-level representations in capacity-limited systems, therefore, but the addressability of information that is the crux of performance in this setting. The formation of elements into objects reduces the unique identifiability of those elements; they are no longer whole entities in themselves, but rather need to be recovered from the object, and there is a performance cost associated with this. Critically, it is the process of object formation that determines the addressability of those sub-object features, not processes of interference *per se*, since all that is required for the information therein to become readily addressable again is for a different object organization to be formed from the acoustic environment. The findings of Bregman and Rudnicky (1975) illuminate the setting shown in **Figure 3** and the different effects of interpolated tones and speech on the ability to judge whether or not the test and standard tones are of the same frequency. When followed by a series of similar sounding tones, the standard is more likely to become bound with those tones into an auditory object than when it is followed by the acoustically distinct speech, therefore the addressability of it as a tone in itself is reduced in the former compared to the latter. We tested this alternative account in a series of experiments (Jones et al., 1997a), two key aspects of which are illustrated in **Figure 5**. In the first case (**Figure 5A**), we compared pitch discrimination performance under conditions where the timing of the tones was the same as that used in the original Deutsch (1970) experiments – that is, the interval between the standard and the first interpolated tone was the same as that between interpolated tones – with conditions where that initial interval was increased. Notice here that the overall interval between standard and test tones has been increased by over a second, so the period over which the comparison has to be made has increased appreciably over the usual form of the task. Nonetheless, this manipulation improves performance significantly. However, the same timing manipulation (not depicted in **Figure 5**) with spoken digits between standard and test tones had no effect on performance, so it is not simply the case that increasing the interval between standard and interpolated material is advantageous to performance; it is only so when such an increase is likely to aid perceptual partitioning of the target material from the interpolated, and such segmentation is already so strongly signified by the change from pure tone to spoken digit that the timing is redundant: the target and the interpolated material have formed two distinct objects regardless of the timing manipulation. In **Figure 5B**, the comparison is between the original type of stimulus and one in which the rate at which interpolated tones are presented is doubled. Again, notice that this means that the number of ostensibly interfering events between standard and test tone has been doubled and yet, this leads to significantly better pitch discrimination performance; an effect diametrically at odds with the classical interference account of the effect of interpolated material. On the other hand, an account framed in terms of the consequences of object formation for the addressability of elements within those objects provides a ready explanation; just as with the manipulation depicted in **Figure 5A**, doubling the rate at which the interpolated tones are presented (**Figure 5B**) leads to them forming a coherent object in themselves, concomitantly leading to the perceptual segmentation of the target tone from that irrelevant material, thereby affording more ready perceptual matching.

It is not easy to see how this set of results can be explained by reference to the decay of or interference with volatile short-term memory representations within limited-capacity storage of individual target events. Increasing the delay and increasing the quantity of interpolated material actually improves performance, and an account framed in terms of the formation of perceptual objects, and the consequences that has for the addressability of information within those objects provides a coherent account, not only of the effects depicted in **Figure 5**, but also of the finding that performance is better when the interpolated material is acoustically different from the target material. Similarity between operationally relevant and irrelevant sources of material affects performance, by this account, not due to processes of interference leading to the degradation of item-level representations of the relevant information, but by affecting the likelihood that relevant information will be bound into a single integrated object along with the irrelevant, coupled with the concomitant loss of individual identity accompanied by such dynamic object formation. The limits to performance, by this account, arise not from burdens on limited capacity systems to store or process task relevant in the presence of task-irrelevant information, but rather on the appropriateness or otherwise of the objects formed in the setting – a function of the nature of the material and the perceptual repertoire of the participant – in the light of the particular requirements of the given task.

Finally, we turn to yet another classic setting in which the limited capacity of short-term retention systems has been investigated – the *suffix effect* (Crowder and Morton, 1969). This refers to the finding that redundant auditory events occurring at the end of a to-be-remembered auditory sequence disrupt serial recall of that sequence, especially recall of items toward the end of that sequence. For example, recall of a random sequence of the digits 1–9 is disrupted if the last digit in the sequence is followed by an auditory item that is not to be recalled (e.g., the spoken word ‘go’). The more acoustically similar the suffix to the list content, the greater its disruptive effect; for example changing the voice between sequence and suffix, changing the location, intensity, timbre and so on, all lead to a decrease in the suffix effect (e.g., Crowder and Morton, 1969; Nairne, 1990; Surprenant et al., 2000). Here again, degradation of representations of the target items by the irrelevant suffix appears to be implicated as the mechanism of disruption. However, it turns out not to be the case. Applying the type of consideration of auditory object formation discussed above, Nicholls and Jones (2002). See also Maidment and Macken, 2012; Maidment et al., 2013) compared the effect on serial recall of a suffix presented on its own, at the end of a to-be-remembered sequence with one presented in the presence of a ‘captor’ sequence running simultaneously with the to-be-remembered sequence of auditory digits. In both types of condition a suffix (the spoken word ‘go’) was presented after the final digit, so according to the item interference account, it should degrade the representation of the final sequence items. However, while the typical disruption to performance occurred under standard conditions, the addition of a captor sequence – repetitions of the word ‘go’ – throughout sequence presentation restored performance, and did so to the extent that the captor sequence formed a coherent perceptual object into which the suffix would be captured. The ability to recall the target information, therefore, is not impeded in the presence of the suffix due to degradation of its representation in limited capacity short-term storage, but rather due to the effect the suffix has, in being bound with the target sequence, of reducing the addressability of information within the sequence, just as illustrated in the examples depicted in **Figures 4** and **5** above. When the suffix, despite occupying the same temporal and acoustic relation to the target material is captured and bound into an object other than the target sequence, then its impact on recall of that sequence is accordingly eliminated.

## Conclusion

Across these diverse settings we see a picture of performance that is not easily captured by an approach that presumes the functional units correspond to ‘items’ – be they tones or speech sounds, relevant or otherwise – and the form of interactions – storage, decay, interference, etc. – amongst these items in limited capacity systems. Rather, performance reflects a much more dynamic set of processes that are implicated in the formation into objects of the whole task environment, the utilization and transformation of those objects, as a function of the nature of the material and the repertoire of the participant, in order to accomplish the particular requirements of a given task. We have described a wide variety of settings in which manipulations, from the classical view of limited capacity short-term memory, would appear subtle or mere matters of input to and output from the limited capacity central system, but which from our dynamic object- oriented approach can be seen as *the* determinants, in combination, of performance.

Importantly, therefore, since they play no explanatory role, there is no requirement within such a framework to determine what the putative static, discrete items might be in terms of which the limits to performance might be quantified – all the explanatory concepts are orthogonal to such concerns. In this way, the reciprocal question of capacity and how it might be expressed or to what it might relate disappears. Further, the approach we have outlined here connotes a different conception of limited performance to that resident within 60 years of cognitive psychology. The analysis of the history and conceptual origins of the cognitive approach with which we began, implicates aspects of a particular *Zeitgeist* in the early conflation of limited performance with limited capacity of the systems underpinning performance. The approach we have outlined here attributes the limited performance which has constituted the focus of so much investigation not to the limited capacity of any system or systems, but rather (we might say, ‘simply’) to the fact that the settings in which such performance has been investigated have, by design, reduced the confluence between the repertoire of the participant, the task she or he is required to accomplish, and the material that forms the focus of that task. In place, then, of capacity and its concomitants, we propose performance limitations be examined by detailed focus on the dynamic interplay of multiple aspects of the task setting. Undoubtedly, these involve aspects of the perceptual-motor systems co-opted to perform a given task. But in themselves these are not determinant, since other aspects of the setting such as the precise requirements of the task, will determine when, what, and if such aspects of the participants’ repertoire impact on performance. This means that there are, in principle, no structural limitations on performance; the more congruent the confluence of the aspects of the setting – the task material, the task requirements and the repertoire of the participant – the better performance will be. The examples we have described here show that such a framework accounts for the modulation of performance in a detailed and dynamic way, as a function of the congruence of multiple aspects of the setting; to the extent that the degree of such congruence is unlimited, then so too is performance.

## Conflict of Interest Statement

The authors declare that the research was conducted in the absence of any commercial or financial relationships that could be construed as a potential conflict of interest.
